# GeneGPT: Augmenting Large Language Models with Domain Tools for Improved Access to Biomedical Information

**Published:** 2023-04-21

**Authors:** Qiao Jin, Yifan Yang, Qingyu Chen, Zhiyong Lu

**Affiliations:** ♣ National Library of Medicine, National Institutes of Health; ♡ University of Maryland, College Park

## Abstract

While large language models (LLMs) have been successfully applied to various tasks, they still face challenges with hallucinations and generating erroneous content. Augmenting LLMs with domain-specific tools such as database utilities has the potential to facilitate more precise and straightforward access to specialized knowledge. In this paper, we present GeneGPT, a novel method for teaching LLMs to use the Web Application Programming Interfaces (APIs) of the National Center for Biotechnology Information (NCBI) and answer genomics questions. Specifically, we prompt Codex (code–davinci–002) to solve the Gene Turing tests with few-shot URL requests of NCBI API calls as demonstrations for in-context learning. During inference, we stop the decoding once a call request is detected and make the API call with the generated URL. We then append the raw execution results returned by NCBI APIs to the generated texts and continue the generation until the answer is found or another API call is detected. Our preliminary results show that GeneGPT achieves state-of-the-art results on three out of four one-shot tasks and four out of five zero-shot tasks in the Gene Turing dataset. Overall, GeneGPT achieves a macro-average score of 0.76, which is much higher than retrieval-augmented LLMs such as the New Bing (0.44), biomedical LLMs such as BioMedLM (0.08) and BioGPT (0.04), as well as other LLMs such as GPT-3 (0.16) and ChatGPT (0.12).

## 1 Introduction

Large language models (LLMs) such as PaLM (Chowdhery et al., 2022) and GPT-4 (OpenAI, 2023) have shown great success on a wide range of general-domain Natural Language Processing (NLP) tasks. They also achieve state-of-the-art performance on many domain-specific tasks like biomedical question answering (Singhal et al., 2022; Liévin et al., 2022; Nori et al., 2023). However, since there is no intrinsic mechanism for auto-regressive LLMs to “consult” with any source of truth, they can generate plausible-sounding but incorrect content (Ji et al., 2023). To tackle the hallucination issue, various studies have been proposed to augment LLMs (Mialon et al., 2023) by either conditioning them on retrieved relevant content (Guu et al., 2020; Lewis et al., 2020; Borgeaud et al., 2022) or allowing them to use other external tools such as program APIs (Parisi et al., 2022; Schick et al., 2023; Qin et al., 2023).

In this work, we propose to teach LLMs to use the Web APIs^1^ of the National Center for Biotechnology Information (NCBI). NCBI provides API access to its biomedical databases and tools including Entrez Programming Utilities (E-utils) and Basic Local Alignment Search Tool (BLAST) URL API (Altschul et al., 1990; Schuler et al., 1996; Sayers et al., 2019). Enabling LLMs to use NCBI Web APIs can provide easier and more precise access to biomedical information, especially for users who are inexperienced with the database systems. The advantage of Web API is to relieve users from implementing functionalities, maintaining large databases, and heavy computation burdens because the only requirement is an internet connection.

We introduce GeneGPT, a novel method that prompts Codex (Chen et al., 2021) to use NCBI Web APIs by in-context learning (Brown et al., 2020). GeneGPT consists of two main modules: (a) a specifically designed prompt that consists of API usage demonstrations, and (b) an inference algorithm that integrates API calls in the Codex decoding process. We evaluate GeneGPT on the Gene Turing dataset (Hou and Ji, 2023), a question answering (QA) benchmark for genomics, and compare it to a variety of other LLMs such as the New Bing^2^, ChatGPT^3^, and BioGPT (Luo et al., 2022). GeneGPT achieves the best performance on three out of four one-shot tasks and four out of five zero-shot tasks, where one instance and no instance are included in the prompt, respectively. It also achieves the second-highest result on the rest tasks. On average, GeneGPT scores 0.76, which is much higher than the previous SOTA (0.44 by New Bing). We hope this pilot study can provide insights for developing systems that improve biomedical information access by integrating LLMs with domain-specific tools such as NCBI Web APIs.

## 2 GeneGPT

In this section, we first introduce the general syntax of NCBI Web APIs (§2.1). We then describe two key components of GeneGPT, including its prompt design (§2.2) and the inference algorithm (§2.3) for downstream tasks.

### 2.1 NCBI Web APIs

We utilize NCBI Web APIs of E-utils that provide access to genomics databases and the BLAST tool for DNA sequence alignment. All Web API calls are implemented by the urllib library in Python.

**E-utils** is the API for accessing the Entrez system (Schuler et al., 1996), a database system that covers 38 NCBI databases of biomedical data (Sayers et al., 2019), such as nucleotide and protein sequences. It provides a fixed URL syntax for rapidly retrieving biomedical information. Specifically, the base URL for an E-utils request is “https://eutils.ncbi.nlm.nih.gov/entrez/eutils/{function}.fcgi”, where function can be esearch, efetch, esummary and etc. esearch returns the unique database identifiers for a given query term, while efetch and esummary return specific information for a given list of identifiers. Important arguments in the URL request include the search term or ids (term or id), the database to use (db), the maximum number of returned items (retmax), and the return format (retmode).

**BLAST URL API** allows users to submit queries to find regions of similarities between nucleotide or protein sequences to existing databases using the BLAST algorithm (Altschul et al., 1990; Boratyn et al., 2013) on NCBI servers. The results can be used to infer relationships between sequences or identify members of gene families. The base URL for the BLAST URL API is “https://blast.ncbi.nlm.nih.gov/blast/Blast.cgi”. By sending different parameters to this API, user can submit and retrieve queries that are executed by NCBI web servers. Every call to the API must include a CMD parameter that defines the type of the call. When submitting queries using CMD=Put, the user can specify the querying database with the DATABASE parameter, the searching program with the PROGRAM parameter, and the query sequence with the QUERY parameter. The user will get an RID after the CMD=Put API call, and can make another API call with the Get command and the returned RID to retrieve its BLAST results. More details can be found in the NCBI BLAST API documentation^4^.

### 2.2 Prompt design

**Figure 1:**
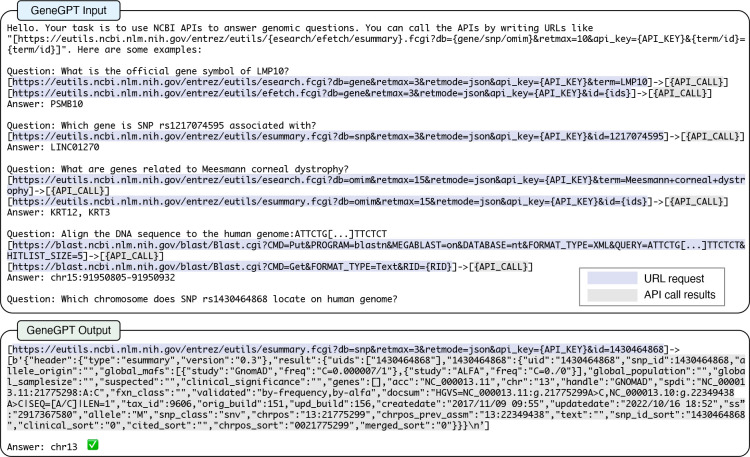
An example of teaching large language models to use NCBI Web APIs and solve genomics questions. The prompt includes three parts: 1. a general task description and an NCBI Web API URL template; 2. Four demonstrations of NCBI API usage (summarized in Table 1); 3. A task-specific test question.

Figure 1 shows an example of our prompt, which consists of three parts. The first two parts are universal across different tasks, and the last part includes a specific test question for inference:

1. The prompt starts with an overall task description (“Your task is to use NCBI APIs to answer genomics questions.”) and the NCBI Web API URL template (described in §2.1).
2. It is followed by four QA instances as demonstrations of using NCBI Web APIs, which are summarized in Table 1. We use them to teach the LLM to use three functions (esearch, efetch, esummary) and three databases (gene, snp, omim) of the NCBI E-utils, as well as the BLAST API. The API URLs and the call results are marked up by “[ ]”, with a special “–>” symbol inserted in between.

**Algorithm 1:** GeneGPT inference algorithm

**Input:** question
**Model:** Codex (code-davinci-002)
**Output:** answer
prompt ← header + demonstrations + question
finished ← False
**while** not finished **do**
next token ← Codex(prompt)
prompt ← prompt + next token
**if** next token is “–>” **then**
# help call Web API
url ← extractLastURL(prompt)
result ← callWebAPI(url)
# append API call result
prompt ← prompt + result
**else if** next token is “\n\n” **then**
answer ← extractAnswer(prompt)
finished ← True
**end if**
**end while**

- The specific test question is then appended to the end of the prompt, with a similar format to the demonstration instances for in-context learning (Brown et al., 2020).

### 2.3 Inference algorithm

The GeneGPT inference algorithm is briefly shown in Algorithm 1. Specifically, we first append the given question to the prompt (described in §2.2) and feed the concatenated text to Codex (code–davinci–002, Chen et al. (2021)) with a temperature of 0. We choose to use Codex for two reasons: (1) it is pre-trained with code data and shows better code understanding abilities, which is crucial in generating the URLs and interpreting the raw API results; (2) its API has the longest (8k tokens) input length among all available models so that we can fit the demonstrations in.

We discontinue the text generation process when the special “–>” symbol is detected, which is the indication for an API call request. Then we extract the last URL and call the NCBI Web API with it. The raw execution results will be appended to the generated text, and it will be fed to Codex to continue the generation. When “\n\n”, an answer indicator used in the demonstrations, is generated, we will stop the inference and extract the answer after the generated “Answer: ”.

## 3 Experiments

### 3.1 The GeneTuring dataset

The Gene Turing dataset (Hou and Ji, 2023) contains 12 tasks, and each task has 50 question-answer pairs. The tasks are classified into four categories: nomenclature, genomics location, functional analysis, and sequence alignment. We use 9 GeneTuring tasks that are related to NCBI resources to evaluate the proposed GeneGPT method. The chosen tasks are briefly described below^5^:

**Nomenclature** is about gene names. We use the gene alias task and the gene name conversion task, where the objective is to find the official gene symbols for their non-official synonyms.

**genomics location** is about the locations of genes, single-nucleotide polymorphism (SNP), and their relations. We include the gene location, SNP location, and gene SNP association tasks. The first two tasks ask for the chromosome locations (e.g., “chr2”) of a gene or an SNP, and the last one asks for related genes for a given SNP.

**Functional analysis** is about gene functions. We use the gene disease association task where the goal is to return related genes for a given disease, and the protein-coding genes task which asks whether a gene is a protein-coding gene or not.

**Sequence alignment** is about DNA sequences. We use the DNA sequence alignment to human genome task and the DNA sequence alignment to multiple species task. The former maps an DNA sequence to a specific human chromosome, while the latter maps an DNA sequence to a specific species (e.g. “zebrafish”).

### 3.2 Compared methods

We compare the proposed GeneGPT method with various baselines evaluated by Hou and Ji (2023), including general-domain GPT-based (Radford et al., 2018) LLMs such as GPT-2 (Radford et al., 2019), GPT-3^6^ (Brown et al., 2020), and ChatGPT^7^, GPT-2-sized biomedical domain-specific LLMs such as BioGPT (Luo et al., 2022) and BioMedLM^8^ (previously known as PubMedGPT), as well as the New Bing^9^, a retrieval-augmented LLM that has access to relevant web pages retrieved by the Bing search engine.

### 3.3 Evaluation

For the performance of the compared methods, we directly use the results reported in Hou and Ji (2023) that are manually evaluated.

To evaluate our proposed GeneGPT method, we follow the general criteria but perform automatic evaluations. Specifically, we only consider *exact* matches between model predictions and the ground truth as correct predictions for all nomenclature and genomics location tasks. For the gene disease association task, we measure the recall as in Hou and Ji (2023) but based on *exact* individual gene matches. For the protein-coding genes task and the DNA sequence alignment to multiple species task, we also consider *exact* matches as correct after applying a simple vocabulary mapping that converts model-predicted “yes”/“no” to “TRUE”/“NA” and Latin species names to their informal names (e.g., “*Saccharomyces cerevisiae*” to “yeast”), respectively. For the DNA sequence alignment to human genome task, we give correct chromosome mapping but incorrect position mapping a score of 0.5 (e.g., chr8:7081648–7081782 v.s. chr8:1207812–1207946), since the original task does not specify a reference genome. Overall, our evaluation of GeneGPT is more strict than the original evaluation of other LLMs in Hou and Ji (2023), which performs manual evaluation and might consider non-exact matches as correct.

### 3.4 Main results

Table 2 shows the performance of GeneGPT on the GeneTuring tasks in comparison with other LLMs. For GeneGPT, four tasks (with “*” in Table 2) are one-shot where one instance is used for API demonstration, and the other five tasks are zero-shot. For the compared LLMs, all tasks are zero-shot.

#### Nomenclature:

GeneGPT achieves state-of-the-art (SOTA) performance on both the one-shot gene alias task (an accuracy of 0.80) and the zero-shot gene name conversion task (an accuracy of 0.98). On average, GeneGPT outperforms New Bing by a large margin (0.89 v.s. 0.76). All other GPT models have accuracy scores of less than 0.10 on the nomenclature tasks.

#### genomics location:

GeneGPT also achieves SOTA performance on all genomics location tasks, including the one-shot gene SNP association task (1.00 accuracy), as well as the zero-shot gene location task (0.62 accuracy) and the zero-shot SNP location task (1.00 accuracy). While the New Bing is comparable to GeneGPT on gene location (0.61 v.s. 0.62), its performance on the two SNP-related tasks is close to 0. Similarly, most other LLMs score less than 0.10.

#### Functional analysis:

New Bing performs better functional analysis tasks than the proposed GeneGPT (average score: 0.91 v.s. 0.58), which is probably because many web pages related to gene functions can be retrieved by the Bing search engine. We also note that other LLMs, especially GPT-3 and ChatGPT, perform moderately well and much better than they perform on other tasks. This might also be due to the fact that many gene-function-related texts are included in their pre-training corpora.

#### Sequence alignment:

GeneGPT performs much better with an average score of 0.65 than all other models including New Bing (0.00), which essentially fails on the sequence alignment tasks. This is not very surprising since sequence alignment is easy with the BLAST tool, but almost impossible for an auto-regressive LLM even with retrieval augmentation as the input sequences are too specific to appear on any web pages.

Although evaluated under a more strict setting (§3.3), GeneGPT achieves a macro-average performance of 0.76 which is much higher than other compared LLMs including New Bing (0.44). Overall, GeneGPT achieves new SOTA performance on three out of four one-shot tasks and four out of five zero-shot tasks and is outperformed by New Bing only on the 2 functional analysis tasks.

## 4 Conclusions

We present GeneGPT, a novel method that teaches large language models to use NCBI Web APIs by in-context learning. Preliminary results show that GeneGPT achieves state-of-the-art performance on 7 GeneTuring tasks, in comparison to various LLMs including New Bing. This indicates that external tools might be superior to relevant web pages for augmenting LLMs to solve genomics questions.

We plan to extend this pilot study with two future directions: (1) fine-tuning LLMs with NCBI API calls instead of in-context learning and (2) exploring multi-hop biomedical question answering (Jin et al., 2022) and chain-of-thought prompting (Wei et al., 2022) to better serve real-life information needs about biomedicine.

## Figures and Tables

**Table 1:** Summary of in-context demonstrations of NCBI Web API usage. We use the QA instances from four tasks: gene alias, gene SNP association, gene disease association, and DNA sequence alignment to human genome (described in §3.1).

#	Task	Database	Function

1	Alias	gene	esearch, efetch
2	Gene SNP	snp	esummary
3	Gene disease	omim	esearch, esummary
4	Alignment	nt	blastn

**Table 2:** Performance of GeneGPT compared to other LLMs on the GeneTuring dataset.

GeneTuring task	GET-2	BioGPT	BioMedLM	GET-3	ChatGPT	New Bing	GeneGPT

**Nomenclature**							
Gene alias*	0.00	0.00	0.04	0.09	0.07	0.66	**0.80**
Gene name conversion	0.00	0.00	0.00	0.00	0.00	0.85	**0.98**
Average	0.00	0.00	0.02	0.05	0.04	0.76	**0.89**

**genomics location**							
Gene SNP association*	0.00	0.00	0.00	0.00	0.00	0.00	**1.00**
Gene location	0.01	0.04	0.12	0.09	0.09	0.61	**0.62**
SNP location	0.03	0.05	0.01	0.02	0.05	0.01	**1.00**
Average	0.01	0.03	0.04	0.04	0.05	0.21	**0.87**

**Functional analysis**							
Gene disease association*	0.00	0.02	0.16	0.34	0.31	**0.84**	0.49
Protein-coding genes	0.00	0.18	0.37	0.70	0.54	**0.97**	0.66
Average	0.00	0.10	0.27	0.52	0.43	**0.91**	0.58

**Sequence alignment**							
DNA to human genome*	0.02	0.07	0.03	0.00	0.00	0.00	**0.44**
DNA to multiple species	0.02	0.00	0.00	0.20	0.00	0.00	**0.86**
Average	0.02	0.04	0.02	0.10	0.00	0.00	**0.65**

**Overall average**	0.00	0.04	0.08	0.16	0.12	0.44	**0.76**

* One-shot learning for GeneGPT, where one instance is included in the prompt for NCBI Web API demonstration. Unlabeled tasks are zero-shot for GeneGPT. **Bolded** numbers denote the highest performance, while underlined numbers denote the second-highest performance.

## Footnotes

1
https://www.ncbi.nlm.nih.gov/home/develop/api/

2
https://www.bing.com/new

3
https://chat.openai.com/

4
https://ncbi.github.io/blast-cloud/dev/api.html

5 We refer our readers to Hou and Ji (2023) for more details.

6 Specifically GPT-3.5 (text-davinci-003).

7 https://chat.openai.com/ (Jan 31 version).

8
https://crfm.stanford.edu/2022/12/15/pubmedgpt.html

9
https://www.bing.com/new
